# Experimental Investigation of Phase Equilibria of the Ho-Ir-O Ternary System at 1073 K

**DOI:** 10.3390/ma16155406

**Published:** 2023-08-01

**Authors:** Viera Homolová, Lucia Čiripová, Ondřej Zobač, Adéla Zemanová, Ladislav Falat

**Affiliations:** 1 Institute of Materials Research, Slovak Academy of Sciences, Watsonova 47, 040 01 Košice, Slovakia; lciripova@saske.sk (L.Č.); lfalat@saske.sk (L.F.); 2 Institute of Physics of Materials, Czech Academy of Sciences, Žižkova 22, 616 62 Brno, Czech Republiczemanova@ipm.cz (A.Z.)

**Keywords:** Ho-Ir-O system, phase diagram, phase equilibria, isothermal section, eutectic microstructure

## Abstract

An experimental study of the phase equilibria of the Ho-rich part of the Ho-Ir-O ternary system at 1073 K by means of x-ray diffraction, differential scanning calorimetry, and scanning electron microscopy has been carried out. Ho-hcp and four binary compounds, namely Ho_3_Ir, Ho_5_Ir_2_, Ho_5_Ir_3_, and Ho_2_O_3_, were identified in the Ho-Ir-O model alloys after long-term annealing (350–1220 h). No solubility of iridium in Ho_2_O_3_ oxide and Ho-hcp was observed. No ternary phase was found. Based on the experimental results, an isothermal section of the Ho-rich part of the Ho-Ir-O system at 1073 K was constructed. In addition, the microstructure of as-cast alloys was studied. An irregular eutectic consisting of faceted Ho-phase in Ho_3_Ir phase was observed in the alloys with Ho-hcp + Ho_3_Ir + Ho_2_O_3_ phase composition, and the temperature of the eutectic reaction Ho-hcp + Ho_3_Ir ↔ liquid was determined.

## 1. Introduction

Holmium and iridium are elements with interesting properties. Iridium is one of the most corrosion-resistant elements, even at very high temperatures [1]. This predisposes iridium and its alloys to use in space applications [2,3] and catalysis [4,5,6,7,8,9]. Iridium and its complexes can also be used in the medical field as therapeutic reagents [10,11,12]. In addition, Ir-Ho bimetallics are suitable for use in nuclear medicine [13]. Holmium is an element with interesting magnetic properties [14]. Thanks to this, the magnetic properties of Ho-Ir compounds [15,16] and Ho-oxide [17,18] are also intensively studied. As for Ir-oxide, it has found application in electrochemistry [19].

Since it is well known that the phase composition of materials determines their properties, studies of phase equilibria and phase diagrams are essential and necessary for the production, research, and development of new materials. No phase diagrams or information about phase equilibria is known either for the Ho-Ir-O ternary system or the binary subsystems Ho-O and Ir-O. For the Ho-Ir system, the speculative phase diagram was drawn by Moffat [20], assuming similarity to the Ce-Ir and La-Ir systems. Later, Okamoto proposed a Ho-Ir phase diagram in the work [21], however with unknown temperatures of phase transformations. In the phase diagram, six intermetallic phases (Ho_3_Ir, Ho_5_Ir_2_, Ho_5_Ir_3_, Ho_3_Ir_2_, HoIr, and HoIr_2_) are presented. The last-mentioned phase is presented with a supposed homogeneity range. With oxygen, the holmium forms only one stable oxide, Ho_2_O_3_ [22]. No other stable oxides are known. Only metastable HoO_1,5_ oxide with cubic lattice cF12 was found by Kashaev et al. [23]. In the Ir-O system, only one oxide, IrO_2_, is known [24]. The crystallographic data of the known phases of the Ho-Ir-O system are summarized in Table 1.

The present work is focused on the experimental study of the microstructure, phases, and phase composition of the Ho-Ir-O alloys after long-term annealing at 1073 K with the aim of contributing to the knowledge of the phase equilibria of the Ho-Ir-O system in the holmium-rich part.

## 2. Materials and Methods

A set of Ho-Ir-O model alloys were produced from high-purity powders of Ho (99.9%), Ho_2_O_3_, and Ir (99.99%). The powders originated from Alfa Aesar (Kandel, Germany). Powders were mixed, pressed into cylindrical compacts, and subsequently melted. The melting was carried out in an argon arc furnace (Mini Arc Melter MAM-1, Bühler, Bodelshausen, Germany) with a tungsten electrode on a water-cooled copper plate in an argon atmosphere of 99.999% purity. A titanium getter was added during the arc-melting stage to remove oxygen. The solidified alloys were remelted several times in order to achieve good homogeneity. The final chemical compositions of the produced alloys (2 g) are shown in Table 2. The alloys were then sealed in evacuated silica glass tubes with titanium chips for oxidation elimination. Subsequently, they were annealed for a long time in an electric resistance furnace (LAC, Židlochovice, Czech Republic) at 1073 K. The specific annealing times are given in Table 2. After the high temperature annealing, the samples were quenched into water to freeze the phases formed during annealing to room temperature and to prevent contact of the hot samples with air.

The samples of as-cast and annealed alloys were studied by scanning electron microscopy (SEM), X-ray diffraction (XRD), and differential scanning calorimetry (DSC).

A scanning electron microscope JEOL JSM-7000F (Jeol, Tokyo, Japan) equipped with a “Thermal field emission gun” (FEG) and INCA energy dispersive X-ray (EDX) analyzer in backscattered electron (BSE) image mode at 15 kV acceleration voltage was used for alloy microstructural characterization and determination of the chemical composition of the alloys and the equilibrium phases.

The identification of stable phases present in the alloys was achieved by XRD. The crystallographic structure of the present phases was adopted from the ICSD database [32]. A smaller part of the samples was ground in a mortar to a fine powder and further used for XRD. The identification of the phases present in the samples was carried out by X-ray powder diffraction using a diffractometer manufactured by EMPYREAN Company (Houston, TX, USA) with CoKα radiation. The measured patterns were interpreted using the High Score Plus SW and ICSD databases by Rietveld analysis. Rietveld refinements of selected diffraction patterns were performed with the automatic feature of the Highscore Plus software.

DSC measurements were performed on the apparatus Netzsch DSC 404C (Netzsch, Selb, Germany). The thermal effect upon heating was measured at a rate of 10 K/min. The experiments were carried out in an inert gas atmosphere (high-purity argon) using a constant gas flow (50 mL/min). The samples were heated in alumina crucibles. The DSC was calibrated using a set of pure metal standards having well-defined melting temperatures (Sn, Al, Zn, Cu, Ag, and Au). Calibration was carried out under the same conditions as the experimental measurements. DSC measurements were used for the determination of the temperature of phase transformations.

**Figure 1:**
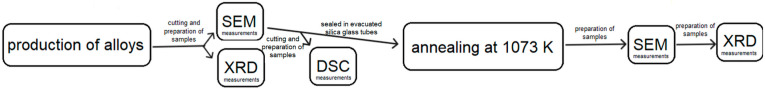
Experiment scheme: SEM—scanning electron microscopy, XRD—X-ray diffraction, DSC—differential scanning calorimetry.

A schematic diagram of experimental work is shown in Figure 1.

## 3. Results and Discussion

### 3.1. Microstructure

**Figure 2:**
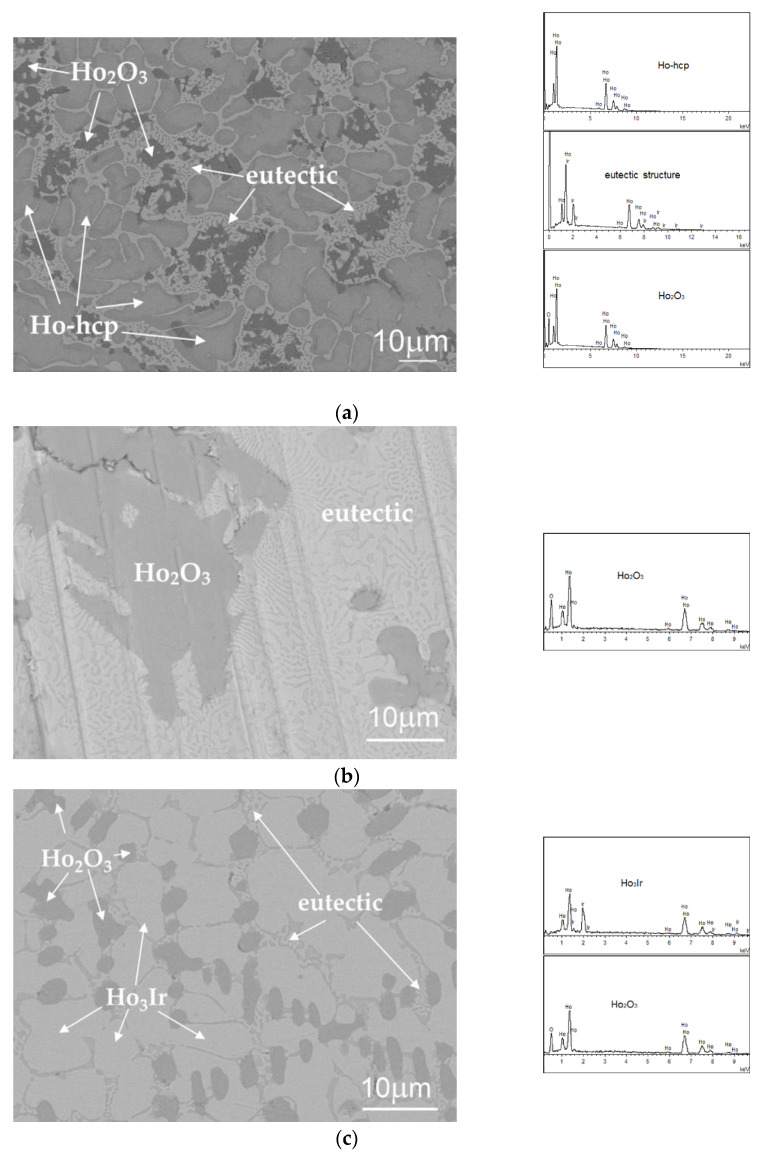

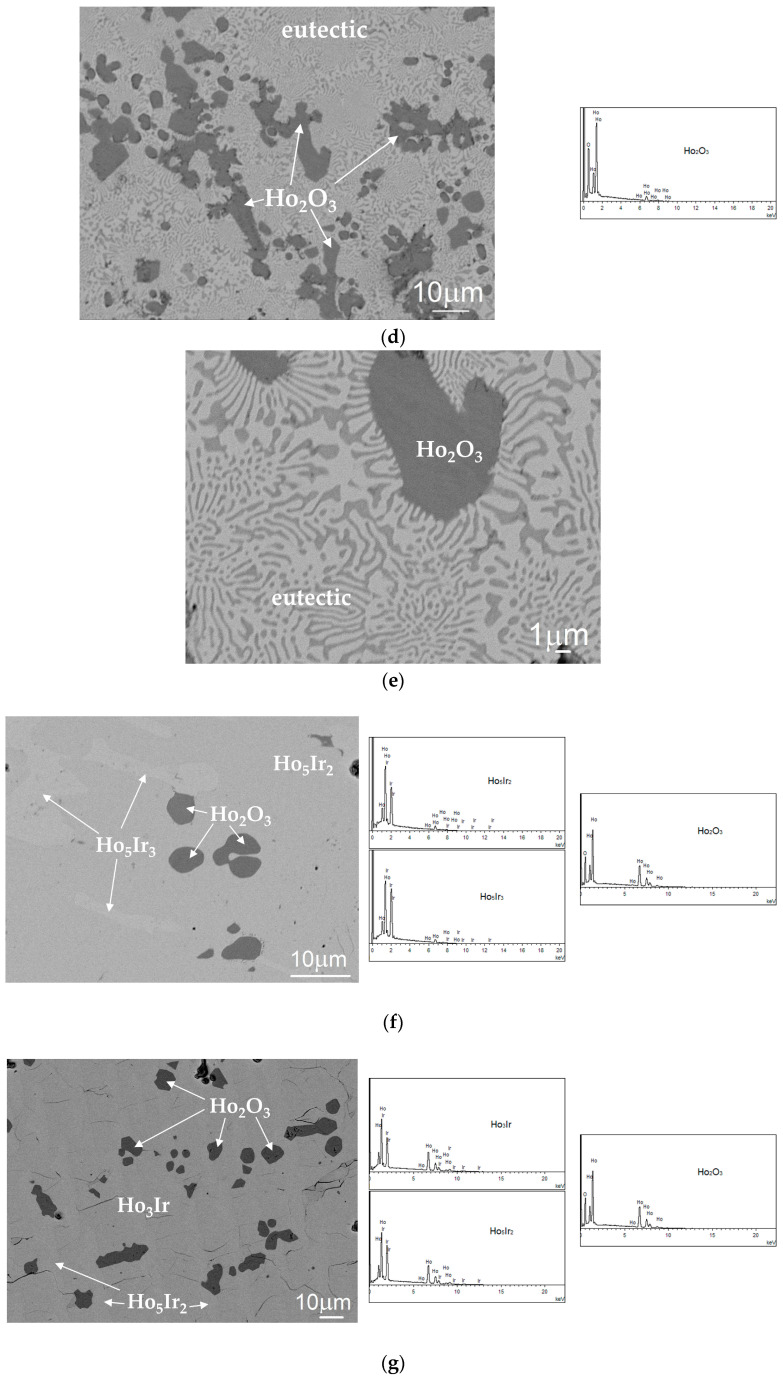
Microstructure of the alloys without annealing with EDX spectra of observed particles (**a**) alloy 1 (67Ho-5Ir-28O at %), (**b**) alloy 2 (57Ho-7Ir-36O at %), (**c**) alloy 3 (59.6Ho-11.4Ir-29O at %), (**d**) alloy 5 (57.5Ho-9Ir-33.5O at %), (**e**) detail of the eutectic in alloy 5 (57.5Ho-9Ir-33.5O at %), (**f**) alloy 6 (55Ho-23Ir-22O at %), and (**g**) alloy 8 (61Ho-19Ir-20O at %).

Figure 2 shows the microstructure of as-cast alloys after production by arc-melting without long-term annealing. The alloys 1, 2, 3, and 5 contain irregular eutectic structures consisting of faceted pure Ho-phase (gray color) in Ho_3_Ir phase (light color); see Figure 2a–e. This observation confirms the eutectic reaction liquid → Ho-hcp + Ho_3_Ir in the binary Ir-Ho-phase diagram suggested by Okamoto [21]. The microstructures of these alloys differ from each other in the amount of eutectic part and the amount and size of other particles. Specifically, the alloy 1, containing the highest amount of holmium among the investigated alloys, contains, in addition to residual eutectic, the dendritic cells of Ho_2_O_3_ and Ho-hcp phases (Figure 2a). The residual eutectic is typically formed in the inter-dendritic space. In alloy 2, the whole space between the particles of Ho_2_O_3_ is completely filled by the eutectic (Figure 2b). No additional Ho-hcp phase particles, besides those in the eutectic, were observed in this alloy. A similar microstructure consisting of Ho_2_O_3_ dendritic cells and irregular eutectic is observed in alloy 5 which has a similar chemical composition as alloy 2 (Figure 2d). Figure 2e shows the eutectic structure of alloy 5 in more detail. The alloy 3, containing the highest amount of iridium among the alloys with the qualitatively same phase composition, contains dendritic cells of the Ho_3_Ir phase, particles of Ho_2_O_3_, and residual eutectic (Figure 2c). The dendritic cells of the Ho_3_Ir phase were not observed in any other alloy with the same phase composition (alloy 1, 2, and 5).

As mentioned above, the eutectic structure of these alloys consist of Ho_3_Ir and Ho-hcp phases. The facet phase (Ho-hcp) is very fine and small, and its identification directly from the EDX spectrum was not possible. The color shade (based on the atomic number contrast) of this phase indicates that it is pure Ho, and from the analysis of the EDX spectrum of the eutectic structure, it can be identified as Ho since there is a lack of oxygen for it to be an oxide. The chemical composition of the eutectic structure (determined as the average composition of the EDX area analysis of the eutectic region) measured in alloy 1 is 83Ho-17Ir in at.%.

**Figure 3:**
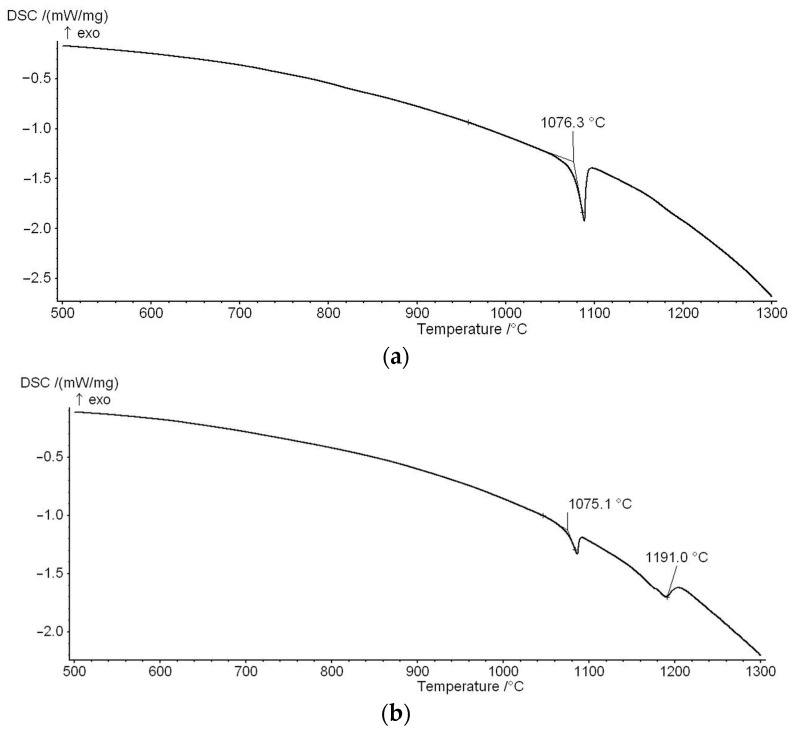
DSC heating curves of the alloys (**a**) alloy 2 (57Ho-7Ir-36O at %), and (**b**) alloy 3 (59.6Ho-11.4Ir-29O at %).

For the as-cast alloys 2 and 3, DSC measurements were performed up to 1300 °C (1573 K), and the heating curves are presented in Figure 3a,b. On the heating curve of the alloy 2, only one phase transformation was noticed in the temperature range of the DSC measurement. The temperature of the phase transformation is 1076.3 °C (1349.45 K). From the analysis of the microstructure, it can be assumed that this temperature represents the temperature of the eutectic reaction Ho-hcp + Ho_3_Ir → liquid. In alloy 3, phase transformation was found at 1075 °C (1348.15 K), and a small endothermic peak on the curve was also identified at 1191 °C (1464.15 K). The lower temperature (1075 °C = 1348.15 K) corresponds to the eutectic temperature, similar to alloy 2. Unlike alloy 2, Ho_3_Ir dendritic cells were observed in alloy 3, so the temperature of 1464.15 K probably corresponds to their formation from the melt (or their melting) in this alloy. Due to the fact that the melting temperature of pure Ho_2_O_3_ is high (2664 K according to [33], 2685 K according to [34]), it is not expected to record the phase transformation related to the melting of oxide by the used method.

The microstructure of alloy 6 (Figure 2f) significantly differs from the microstructures of the previously mentioned alloys. The microstructure of this alloy is three-phase, characterized by the presence of small rounded dark-gray Ho_2_O_3_ oxides and larger light-gray particles of Ho_5_Ir_3_ phase in the gray matrix (Ho_5_Ir_2_), as is seen in Figure 2f. The alloy does not contain eutectic. Similar microstructure is observed also in the alloy 4.

The small dark-gray-colored appearing particles of Ho_2_O_3_ and the striated structure of light-gray-colored appearing Ho_5_Ir_2_ and medium-gray-colored HoIr_3_ phases are present in alloy 8 (Figure 2g).

Metastable HoO_1,5_ phase with the cubic CaF_2_-type structure, founded in the Ho-O system by Kaschaev et al. [23], was not found in any as-cast Ho-Ir-O alloys. The XRD measurements of the investigated alloys confirmed the cubic Mn_2_O_3_ type structure of the found Ho_2_O_3_ oxide.

### 3.2. Phase Analysis of Equilibrated Alloys

**Figure 4:**
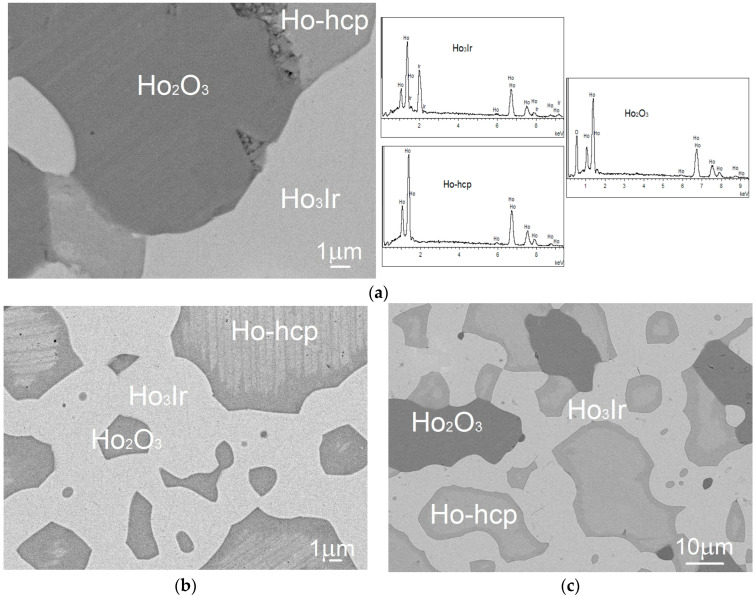

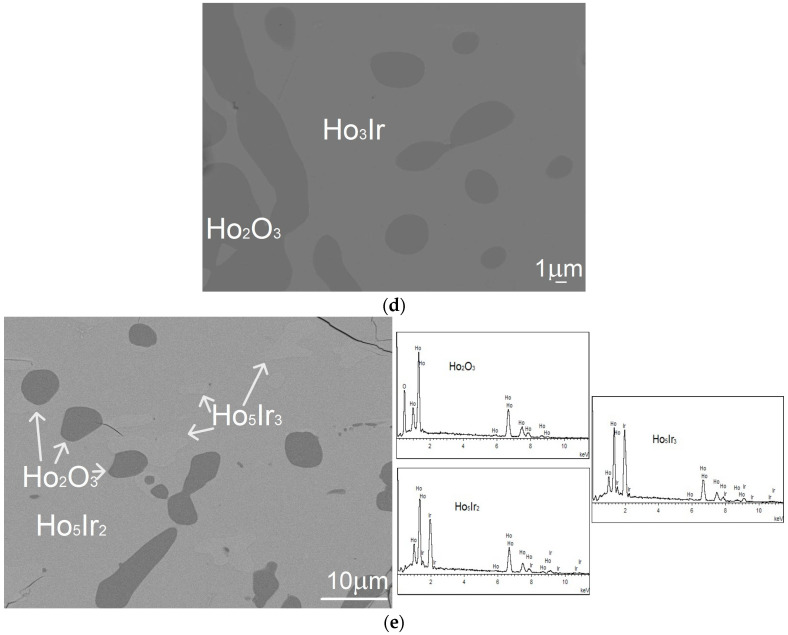
Microstructure of the alloys after annealing at 1073 K: (**a**) alloy 1 (67Ho-5Ir-28O at %) with EDX spectra of identified phases; (**b**) alloy 2 (57Ho-7Ir-36O at %); (**c**) alloy 5 (57.5Ho-9Ir-33.5O at %); (**d**) alloy 7 (61Ho-16Ir-23O at %), and (**e**) alloy 6 (55Ho-23Ir-22O at %) with EDX spectra of identified phases.

**Figure 5:**
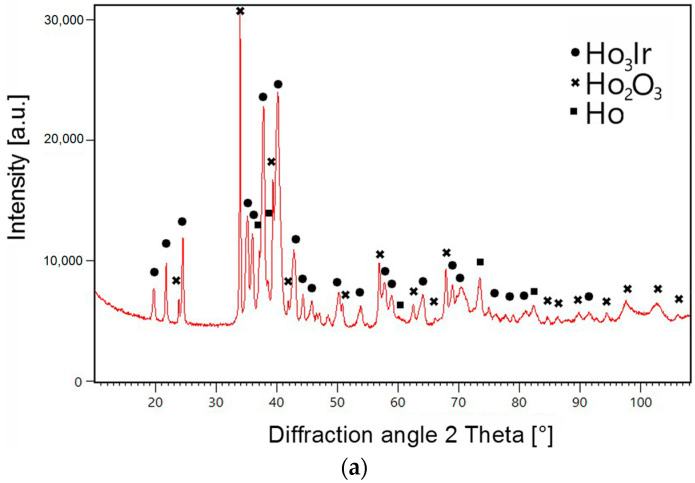

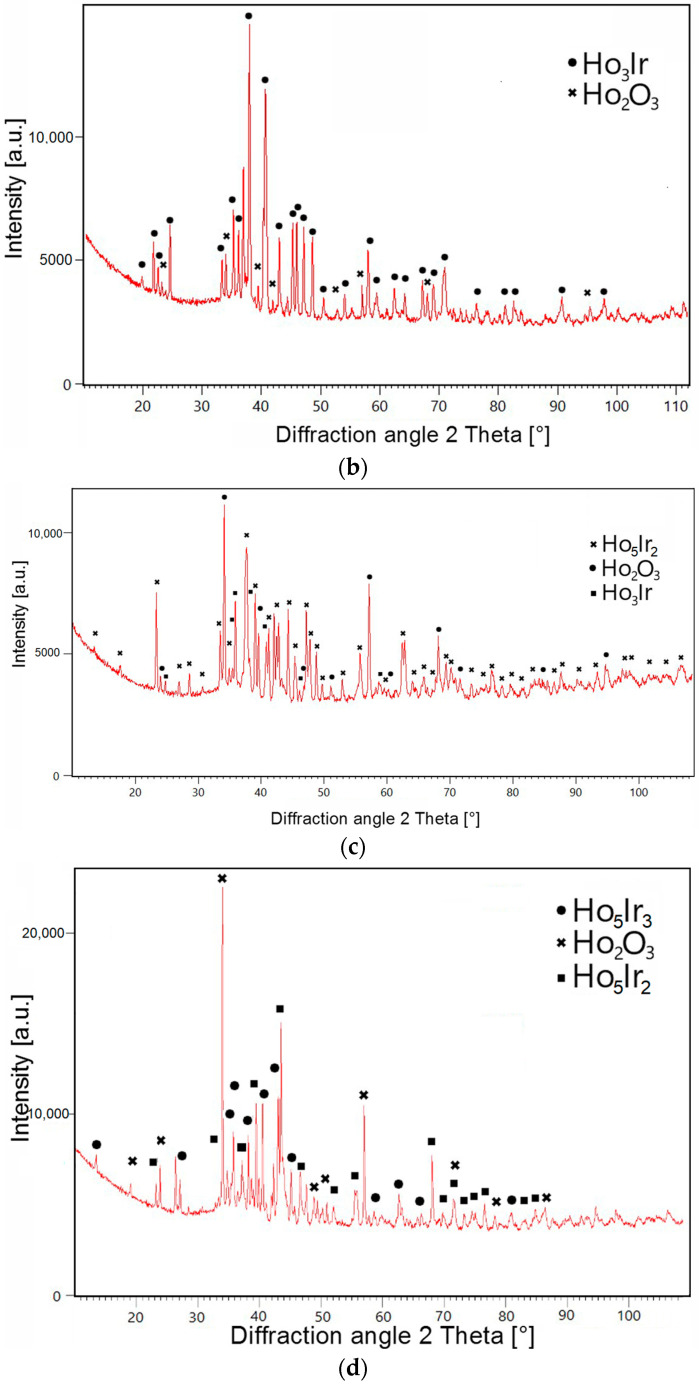
XRD patterns of the investigated alloys: (**a**) alloy 3 (59.6Ho-11.4Ir-29O at %); (**b**) alloy 7 (61Ho-16Ir-23O at %); (**c**) alloy 8 (61Ho-19Ir-20O at %); and (**d**) alloy 4 (55Ho-17Ir-28O at %).

The microstructure of the selected alloys after annealing at 1073 K is shown in Figure 4a–e. Figure 5 shows the XRD patterns of the selected alloys. The identified equilibrium phases in the investigated alloys are listed in Table 3.

A three-phase structure consisting of holmium with hcp structure, the Ho_3_Ir phase, and Ho_2_O_3_ oxide was found in the alloys 1 (Figure 4a), 2 (Figure 4b), 3, and 5 (Figure 4c). The darkest particles correspond to Ho_2_O_3_ oxide, the gray particles correspond to holmium, and the lightest color represents the intermetallic compound Ho_3_Ir. For alloy 1, the EDX spectra of identified phases are also shown in Figure 4a. The three-phase structure of alloy 3 is presented by the XRD pattern in Figure 5a.

Two-phase equilibrium Ho_3_Ir + Ho_2_O_3_, shown in Figure 4d, was identified in alloy 7. The XRD pattern of the alloy is shown in Figure 5b.

The XRD pattern in Figure 5c shows three-phase equilibrium Ho_2_O_3_ + Ho_3_Ir + Ho_5_Ir_2_ founded in alloy 8.

Three equilibrium phases, namely Ho_2_O_3_ oxide (the darkest particles) and Ho_5_Ir_2_ and Ho_5_Ir_3_ (the lightest particles) intermetallic compounds, were observed in the alloys 6 (Figure 4e) and 4 (Figure 5d). The EDX spectra of identified phases in alloy 6 are shown in Figure 4e.

No iridium solubility was noticed in the Ho-hcp phase by using experimental methods, which is consistent with the Ho-Ir binary phase diagram suggested by Okamoto [21]. Similarly, no iridium solubility was observed in Ho_2_O_3_ oxide. A small amount of oxygen was observed in intermetallic compounds as is seen also in EDX spectra (Figure 3a,e). However, the ratios of holmium and iridium correspond to the stoichiometric composition of the phases: 3:1 for Ho_3_Ir, 5:2 for Ho_5_Ir_2_, and 5:3 for Ho_5_Ir_3_. It indicates the interstitial solubility of oxygen in the binary Ho-Ir compounds.

From XRD measurements of alloys 2 and 4, the lattice parameters of all equilibrium phases were determined. The obtained parameters are shown in Table 4. Most of the lattice parameters of the studied phases have higher values compared to the lattice parameters of pure binary phases and Ho listed in Table 1. It is related to the temperature dependence of the lattice parameters and possibly also to the solubility of the third element in the binary phases. Parameters for Ho-hcp in Table 1 are determined at 300 K [25], and nonlinear dependence was found for this phase [35]. The lattice parameters of the Ho_2_O_3_ phase determined for alloys 2 and 4 are very similar, which is expected since they were measured at the same temperature and the solubility of iridium in this phase was not observed. Only the determined parameters for the Ho_5_Ir_3_ phase (Table 4) are lower than the literature ones [29], see Table 1, but these literature parameters were determined at a higher temperature (1173 K). It can therefore be concluded that all determined parameters are in accordance with data from the literature, taking into account the temperature dependence of the parameters and the solubility of oxygen in the binary Ir-Ho-phases.

### 3.3. Isothermal Section

**Figure 6:**
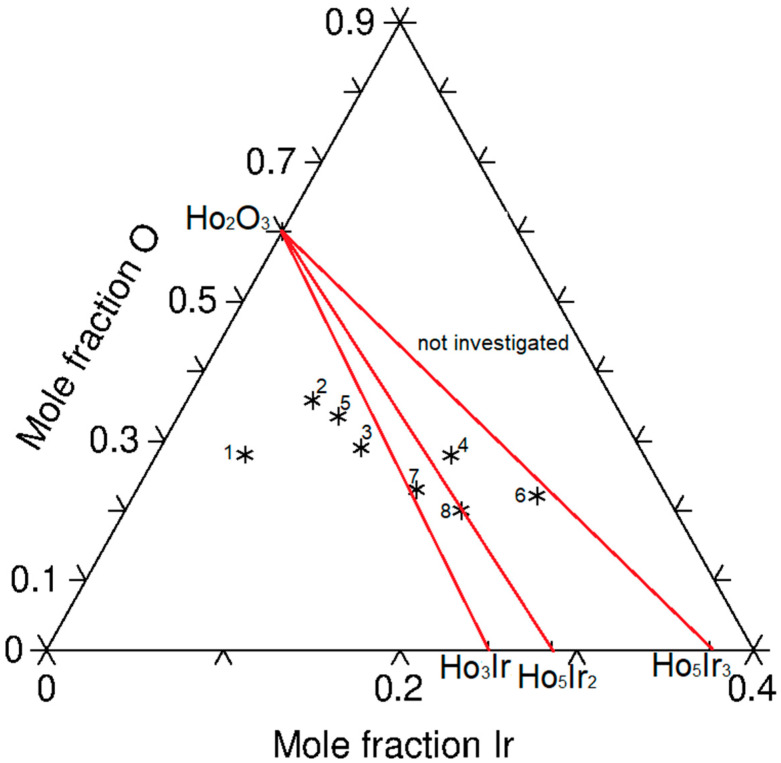
The suggested Ho-rich part of the isothermal section of the Ho-Ir-O phase diagram at 1073 K. The composition of the investigated alloys is marked by stars.

Based on the obtained experimental results for equilibrated alloys, the Ho-rich part of the isothermal section of the Ho-Ir-O phase diagram at 1073 K was constructed; see Figure 6. Three three-phase fields are determined in this part of the phase diagram, namely Ho-hcp + Ho_3_Ir + Ho_2_O_3_, Ho_3_Ir + Ho_2_O_3_ + Ho_5_Ir_2_ and Ho_2_O_3_ + Ho_5_Ir_2_ + Ho_5_Ir_3_. No ternary compound was found in the Ho-rich part of the ternary system at 1073 K. Oxygen identified in intermetallic phases was not taken into account when constructing the diagram. The part of the diagram on the right side of the two-phase region Ho_2_O_3_ + Ho_5_Ir_3_ (Figure 6) has not been investigated experimentally.

Since there are no other experimental results known in the literature regarding the studied Ho-Ir-O ternary system, no data comparison can be made at this point in our investigation.

## 4. Summary and Conclusions

The phase equilibria of the Ho-rich part of the Ho-Ir-O ternary system at 1073 K were investigated experimentally. The results can be summarized as follows:

- The as-cast alloy’s microstructure was studied. An irregular eutectic consisting of faceted Ho-phase in Ho_3_Ir phase was observed in the microstructure of alloys with Ho-hcp + Ho_3_Ir + Ho_2_O_3_ phase composition. The temperature of the eutectic reaction Ho-hcp + Ho_3_Ir ↔ liquid was determined.
- No iridium solubility was observed in the Ho-hcp phase or in Ho_2_O_3_ oxide.
- The Ho-rich part of the isothermal section of the Ho-Ir-O phase diagram at 1073 K was suggested. A total of three three-phase regions were observed in this part of the ternary system and no ternary compound was found. The existence of four binary compounds, Ho_3_Ir, Ho_5_Ir_2_, Ho_5_Ir_3_, and Ho_2_O_3_, was confirmed in this part of the ternary system at 1073 K. The region of the phase diagram with higher amounts of iridium and oxygen was not investigated.

## Figures and Tables

**Table 1:** Crystallographic data of the known stable phases of the Ho-Ir-O system.

System	Phases	Pearson Symbol	Space Group	Lattice Parameters [nm]	Reference
Ho-Ir	Ho-hcp	hP2	P6_3_/mmc	a = 0.3576 c = 0.56136	[25]
	HoIr	cP2	Pm3m	a = 0.3383	[26]
	HoIr_2_	cF24	Fd3m	a = 0.7490	[27]
	Ho_3_Ir	oP16	Pnma	a = 0.7186 b = 0.9139 c = 0.6326	[28]
	Ho_3_Ir_2_	tI140	I4/mcm	a = 1.1132 c = 2.492	[29]
	Ho_5_Ir_2_	mC28	C2/c	a = 1.5497 b = 0.63399 c = 0.7169 β = 96.74°	[30]
	Ho_5_Ir_3_	hP16	P6_3_/mcm	a = 1.0822 c = 0.6255	[29]
	Ir	cF4	Fm3m	a = 0.38385	[25]
Ho-O	Ho_2_O_3_	cI80	Ia3	a = 1.06078	[22]
Ir-O	IrO_2_	tp6	P4_2_/mnm	a = 0.44990 c = 0.31546	[31]

**Table 2:** Chemical composition and condition of annealing of alloys.

Alloy	Composition [at %]	Conditions of Annealing [K/h]
1	67Ho-5Ir-28O	1073/350
2	57Ho-7Ir-36O	1073/350
3	59.6Ho-11.4Ir-29O	1073/350
4	55Ho-17Ir-28O	1073/350
5	57.5Ho-9Ir-33.5O	1073/1220
6	55Ho-23Ir-22O	1073/735
7	61Ho-16Ir-23O	1073/735
8	61Ho-19Ir-20O	1073/350

**Table 3:** Experimentally identified equilibrium phases in the investigated alloys after annealing at 1073 K.

Alloy	Identified Phases
1	Ho-hcp, Ho_3_Ir, Ho_2_O_3_
2	Ho-hcp, Ho_3_Ir, Ho_2_O_3_
3	Ho-hcp, Ho_3_Ir, Ho_2_O_3_
4	Ho_5_Ir_2_, Ho_5_Ir_3_, Ho_2_O_3_
5	Ho-hcp, Ho_3_Ir, Ho_2_O_3_
6	Ho_5_Ir_2_, Ho_5_Ir_3_, Ho_2_O_3_
7	Ho_3_Ir, Ho_2_O_3_
8	Ho_3_Ir, Ho_2_O_3,_ Ho_5_Ir_2_

**Table 4:** Lattice parameters of identified phases determined from X-ray measurements.

Alloy	Phase	Lattice Parameters [nm]
2	Ho-hcp	a = 0.3581122 c = 0.5637031
2	Ho_3_Ir	a = 0.7198528 b = 0.9185608 c = 0.6342015
2	Ho_2_O_3_	a = 1.0610020
4	Ho_2_O_3_	a = 1.0610090
4	Ho_5_Ir_2_	a = 1.56523 b = 0.637546 c = 0.7252621 β = 97.78°
4	Ho_5_Ir_3_	a = 1.080829 c = 0.625656

## Data Availability

Not applicable.
